# Novel opportunities for clinical pharmacy research: development of a machine learning model to identify medication related causes of delirium in different patient groups

**DOI:** 10.1007/s11096-024-01707-z

**Published:** 2024-04-09

**Authors:** Anita Elaine Weidmann, Edward William Watson

**Affiliations:** 1 Department of Clinical Pharmacy, Institute of Pharmacy, Innsbruck University, Innrain 80, 6020 Innsbruck, Austria; 2 Department of Media and Learning Technology, Innsbruck University, Innrain 52, 6020 Innsbruck, Austria

**Keywords:** Artificial intelligence, Clinical pharmacy information systems, Delirium, Drug prescribing, Machine intelligence, Patient safety

## Abstract

The advent of artificial intelligence (AI) technologies has taken the world of science by storm in 2023. The opportunities of this easy to access technology for clinical pharmacy research are yet to be fully understood. The development of a custom-made large language model (LLM) (DELSTAR) trained on a wide range of internationally recognised scientific publication databases, pharmacovigilance sites and international product characteristics to help identify and summarise medication related information on delirium, as a proof-of-concept model, identified new facilitators and barriers for robust clinical pharmacy practice research. This technology holds great promise for the development of much more comprehensive prescribing guidelines, practice support applications for clinical pharmacy, increased patient and prescribing safety and resultant implications for healthcare costs. The challenge will be to ensure its methodologically robust use and the detailed and transparent verification of its information accuracy.

## Introduction

With the advent of artificial intelligence (AI) models, for use by the public dominating the headlines in 2023, a fierce debate ensued about their “appropriate and inappropriate use” in academic research [1]. While the discussions mainly centre around the ethical implications of the academic writing skills of such generative AI models, it raises the question what novel opportunities generative AI presents for clinical pharmacy research [2]. As clinical pharmacy practice operates in a complex interdisciplinary healthcare system, clinical pharmacy practice research is equally complex and transdisciplinary, potentially opening up a lot of opportunity for bridging the disciplines using artifical intelligence in new ways [3]. One such potential application for artificial intelligence is the development of delirium prescribing resources across different patient groups.

### Delirium as a multifactorial syndrome

Delirium is an acute disturbance in attention and cognition that is associated with significant functional decline, distress, and increased mortality [4]. It impacts up to 30% of hospitalized adults most commonly affecting patients with advanced age, cognitive decline, and medical or surgical comorbidity rising to 50–70% in mechanically ventilated patients [5, 6]. Delirium increases healthcare costs by 50% among those diagnosed with delirium superimposed on dementia (DSD) [7]. While the pathophysiology remains poorly understood, the underlying cause of delirium can involve anything that stresses the baseline homeostasis such as substance intoxication or withdrawal, medication side effects, infection, surgery, metabolic derangements, pain, or common conditions such as constipation or urinary retention [8] with 33 predisposing and 112 precipitating factors across all settings having been identified [9].

Medication-associated factors are both noted as predisposing and precipitating however, no further detail is provided that would allow the correct dosing, management, and prevention of these medication effects [10]. Published literature provides evidence that any medication that increases the anticholinergic burden, exhibits a sedative- or antimuscarinic property; precipitates a serotonin syndrome or sleep disturbances is thought to carry a substantial (cumulative) risk [11]. In addition, age related pharmacokinetic and pharmacodynamic changes as well as age related kidney and liver changes presents a risk due to the changes in drug metabolism and a possible increase in blood–brain barrier permeability [12]. To date there is no comprehensive documentation detailing the risk and use of drugs and drug classes associated with delirium providing a prescribing decision aid for clinicians. The generation of such a prescribing decision aid is the aim of the research.

### Evolution of LLMs

With the advent of natural language processing (NLP) and machine learning (ML) models in healthcare since the late 1990s [13], delirium risk can now be predicted with 94.1% accuracy in geriatric internal medicines inpatients [14]. Building accurate predictive models based on logistic regression algorithm is widely used in the medical field [15] with Generative AI now offering an opportunity to augment and accompany the output compared against these logistic regression models [16, 17]. As a result, several logistic regressions, machine learning-based delirium prediction models have been developed since 2018 [18–24]. The minority however include medication as a predictive variable because the process of identifying predictors from among the multitude of medications in various combinations, formulations and dosages would be very complex to capture [25].

### Development of a large language model (LLM) to identify medication related causes

**Fig. 1:**
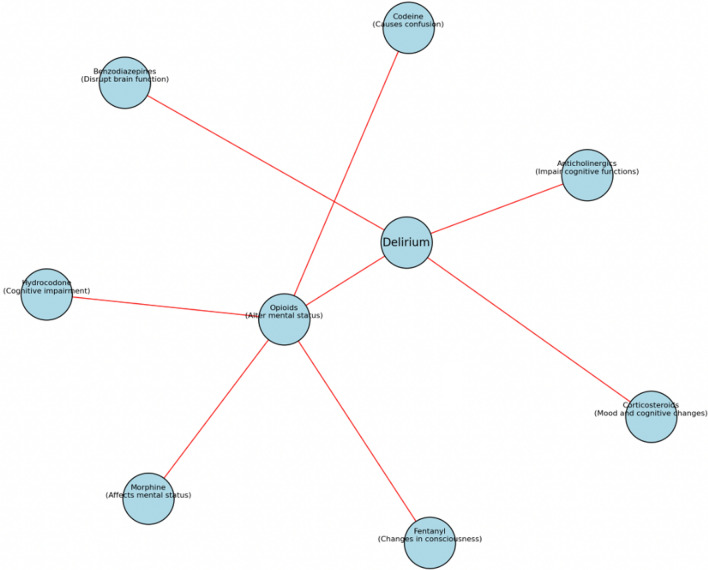
An example of what the output node graph generated by the DELSTAR AI tool may look like. Each node represents a drug class, with the ambition that each node can be further explored to detail drugs, context (symptoms, mechanism of action etc.) and source reference(s) associated with delirium. In this example, the ‘Opioid’ node has been expanded further showing different opioid drugs and key symptom associated with delirium

To support the development of a comprehensive medication-related prescribing decision support application, we built a delirium specific customised large language model (LLM) based tool focused on medication related information (named DELSTAR) as a proof-of-concept. The model uses the custom function of a generative pre-trained transformer (GPT) made available by Open AI (vs.4), also known as GPT4. The proof-of-concept DELSTAR AI tool targets collections of peer reviewed papers as a source of data, using application program interfaces (APIs) where possible. APIs are a means by which one website can talk to another in a program friendly text-based data format. Plain text queries are formed, submitted and results are passed as semantic visualisations for the researcher to navigate in their pathway of inquiry. In other words, the researcher “asks” using normal language, the AI reaches out via the APIs and then “answers” using a visual map. The targeted sources include 39 internationally recognised scientific publication databases, two internationally recognised pharmacovigilance databases and two international databases detailing product characteristics. The information is presented in the form of an interactive Node Graph. Each node representing e.g. a drug class with the ambition that these can be further explored detailing drugs, context and source reference. An example of what the output node graph generated by the DELSTAR AI tool may look like is given in Fig. 1. As the information published across the 39 scientific databases is used as the source data it may be possible to identify semantic patterns between medication and other precipitating and predisposing factors as published by Ormseth (2023) [9]. Correlation to specific patient and medication related factors such as timing to exposure of medicine is limited to the reporting on the pharmacovigilance databases only. The customisation of GPT4 into DELSTAR took a matter of hours; its validation, however, will take much longer. Validation will determine how well the model captures and accurately reflects relevant information from the literature using the creation of a validation dataset, error analysis, consistency, sensitivity and specificity. Following which the model will be fine-tuned. Feedback from medical prescribers across a wide range of specialities will present the final validation step to assess its accuracy and comprehensiveness of information, relevance to practice and utility [26].

The output generated by DELSTAR will be compared against the information gathered across several state-of-the-art systematic reviews [submitted for publication] and the quality, completeness and accuracy of its information assessed. We are curious to learn if this approach has the potential to augment the current state-of-the-art systematic literature review process by providing the researcher with an additional semantic method of information gathering.

## Implications and future directions

This proof-of-concept delirium tool shows how the advent of easily accessible generative artificial intelligence (GenAI) technology has the potential to augment clinical pharmacy practice research with relative ease to help manage and identify medication related patterns across large volumes of data [27]. This holds great promise for the development of much more comprehensive prescribing guides, practice support applications for clinical pharmacy, increased patient and prescribing safety and resultant implications for healthcare costs [28, 29]. The challenge for clinical pharmacy practice research will be to ensure its methodologically robust use and the detailed and transparent verification of its information accuracy in close collaboration with data and information-technology scientists [30].

## References

[1] Weidmann AE. Artificial intelligence in academic writing and clinical pharmacy education: consequences and opportunities. Int J Clin Pharm. 2024. 10.1007/s11096-024-01705-1 10.1007/s11096-024-01705-1 PMC11133206 38472596

[2] Levivien C, Cavagna P, Grah A, et al. Assessment of a hybrid decision support system using machine learning with artificial intelligence to safely rule out prescriptions from medication review in daily practice. Int J Clin Pharm. 2022;44(2):459–65. 10.1007/s11096-021-01366-4.. 34978662 10.1007/s11096-021-01366-4

[3] Nazar Z, Mohammad Naseralallah L, Stewart D, et al. The use of behavioural theories, models, and frameworks in pharmacy practice research: a scoping review. Int J Clin Pharm. 2024. 10.1007/s11096-023-01674-x. 38175323 10.1007/s11096-023-01674-x PMC11133055

[4] Hasieh TT, Inouye S, Oh ES. Delirium in the elderly. Clin Geriatr Med. 2020;36(2):183–99. 10.1016/j.cger.2019.11.001. 32222295 10.1016/j.cger.2019.11.001

[5] Ospina JP, King IVF, Madva E, et al. Epidemiology, mechanisms, diagnosis, and treatment of delirium: a narrative review. Clin Med Ther. 2018;1(1):3. 10.24983/scitemed.cmt.2018.00085. 10.24983/scitemed.cmt.2018.00085

[6] Wilson JE, Mart MF, Cunningham C, et al. Delirium. Nat Rev Dis Primers. 2020;6:90. 10.1038/s41572-020-00223-4. 33184265 10.1038/s41572-020-00223-4 PMC9012267

[7] Kinchin I, Mitchell E, Agar M, et al. The economic cost of delirium: a systematic review and quality assessment. Alzheimers Dement. 2021;17:1026–41. 10.1002/alz.12262. 33480183 10.1002/alz.12262

[8] Lourdes Ramirez Echeverria M, Schoo C, Paul M. *StatPearls*: Delirium. 2022. https://www.ncbi.nlm.nih.gov/books/NBK470399/. Accessed 28 Feb 2024.

[9] Ormseth CH, LaHue SC, Oldham MA, et al. Predisposing and precipitating factors associated with delirium: a systematic review. Jama Netw Open. 2023;6(1): e2249950. 10.1001/jamanetworkopen.2022.49950. 36607634 10.1001/jamanetworkopen.2022.49950 PMC9856673

[10] Day C, Manning K, Abdullah F, et al. Delirium in HIV-infected patients admitted to acute medical wards post universal access to antiretrovirals in South Africa. S Afr Med J. 2021;111(10):974–80. 34949292 10.7196/SAMJ.2021.v111i10.15628

[11] Katipoğlu B, Demircan S, Naharcı M. Association of drug burden index on delirium in community-dwelling older adults with dementia: a longitudinal observational study. Int J Clin Pharm. 2023;45:1267–76. 10.1007/s11096-023-01551-7. 36933080 10.1007/s11096-023-01551-7

[12] Mangoni AA, Jackson SH. Age-related changes in pharmacokinetics and pharmacodynamics: basic rinciples and practical applications. Br J Clin Pharmacol. 2004;57(1):6–14. 10.1046/j.1365-2125.2003.02007.x. 14678335 10.1046/j.1365-2125.2003.02007.x PMC1884408

[13] Inouye S, Viscoli C, Horwitz R, et al. A predictive model for delirium in hospitalised elderly medical patients based on admission characteristics. Ann Intern Med. 1993;119:474–81. 10.7326/0003-4819-119-6-199309150-00005. 8357112 10.7326/0003-4819-119-6-199309150-00005

[14] Li Q, Zhao Y, Chen Y, et al. Developing a machine learning model to identify delirium risk in geriatric internal medicine inpatients. Eur Geriatr Med. 2022;66:1–11. 10.1007/s41999-021-00562-9. 10.1007/s41999-021-00562-9 34553310

[15] Liao L, Mark DB. Clinical prediction models: Are we building better mousetraps? J Am Coll Cardiol. 2003;42:851–3. 10.1016/S0735-1097(03)00836-2. 12957431 10.1016/S0735-1097(03)00836-2

[16] Wuest T, Weimer D, Irgens C, et al. Machine learning manufacturing: advantages, challenges and applications. Prod Manuf Res. 2016;4(1):23–45. 10.1080/21693277.2016.1192517. 10.1080/21693277.2016.1192517

[17] An Q, Rahman S, Zhou J, et al. A comprehensive review on machine learning in healthcare industry: classification, restrictions, opportunities and challenges. Sensors (Basel). 2023;23(9):4178. 10.3390/s23094178. 37177382 10.3390/s23094178 PMC10180678

[18] Wong A, Young AT, Liang AS, et al. Development and validation of an electronic health record-based machine learning model to estimate delirium risk in newly hospitalized patients without known cognitive impairment. JAMA Netw Open. 2018;1(4): e181018. 10.1001/jamanetworkopen.2018. 30646095 10.1001/jamanetworkopen.2018.1018 PMC6324291

[19] Corradi JP, Thompson S, Mather JF, et al. Prediction of incident delirium using a random forest classifier. J Med Syst. 2018;42(261):1–10. 10.1007/S10916-018-1109-0. 10.1007/S10916-018-1109-0 30430256

[20] Oh J, Cho D, Park J, et al. Prediction and early detection of delirium in the intensive care unit by using heart rate variability and machine learning. Physiol Meas. 2018;39: 035004. 10.1088/1361-6579/AAAB07. 29376502 10.1088/1361-6579/AAAB07

[21] Hercus C, Hudaib A-R. Delirium misdiagnosis risk in psychiatry: a machine learning-logistic regression predictive algorithm. BMC Health Serv Res. 2020;20(151):1–7. 10.1186/S12913-020-5005-1. 10.1186/S12913-020-5005-1 PMC7045404 32106845

[22] Davoudi A, Ozrazgat-Baslanti T, Ebadi A, et al. Delirium prediction using machine learning models on predictive electronic health records data. In: Proceedings—2017 IEEE 17th international conference on bioinformatics and bioengineering, BIBE 2017. 2017. p. 568–73. 10.1109/BIBE.2017.00014. 10.1109/BIBE.2017.00014 PMC6211171 30393788

[23] Racine AM, Tommet D, D’Aquila ML, et al. Machine learning to develop and internally validate a predictive model for post-operative delirium in a prospective, observational clinical cohort study of older surgical patients. J Gen Intern Med. 2021;36:265–73. 10.1007/S11606-020-06238-7. 33078300 10.1007/S11606-020-06238-7 PMC7878663

[24] Nabeel H, Hirsch GM, Abidi SR, et al. Exploiting machine learning algorithms and methods for the prediction of agitated delirium after cardiac surgery: models development and validation study. JMIR Med Inform. 2019;7(4):E14993. 10.2196/14993. 31558433 10.2196/14993 PMC6913743

[25] Racine AM, Tommet D, D’Aquila ML, et al. Machine learning to develop and internally validate a predictive model for post-operative delirium in a prospective, observational clinical cohort study of older surgical patients. J Gen Intern Med. 2021;36:65–273. 10.1007/s11606-020-06238-7. 10.1007/s11606-020-06238-7 PMC7878663 33078300

[26] Corradi J, Thompson S, Mather J, et al. Prediction of incident delirium using a random forest classifier. J Med Sys. 2018;42(261):1–10. 10.1007/s10916-018-1109-0. 10.1007/s10916-018-1109-0 30430256

[27] Inouye S, Dyck C, Alessi C, et al. Clarifying confusion: the confusion assessment method. A new method for detection of delirium. Ann Intern Med. 1990;113(12):941–8. 10.7326/0003-4819-113-12-941. 2240918 10.7326/0003-4819-113-12-941

[28] Cole M. Delirium in elderly patients. Am J Geriatr Psychiatry. 2004;12(1):7–21. 10.1097/00019442-200401000-00002. 14729554 10.1097/00019442-200401000-00002

[29] Roosan D, Chok J, Baskys A, et al. PGxKnow: a pharmacogenomics educational HoloLens application of augmented reality and artificial intelligence. Pharmacogenomics. 2022;23(4):235–45. 10.2217/pgs-2021-0120. 35083917 10.2217/pgs-2021-0120

[30] Ranchon F, Chanoine S, Lambert-Lacroix S, et al. Development of artificial intelligence powered apps and tools for clinical pharmacy services: a systematic review. Int J Med Inform. 2023;172: 104983. 10.1016/j.ijmedinf.2022.104983. 36724730 10.1016/j.ijmedinf.2022.104983

